# Amplification Free Detection of SARS-CoV-2 Using Multi-Valent Binding

**DOI:** 10.1021/acssensors.2c01340

**Published:** 2022-12-09

**Authors:** Appan Roychoudhury, Rosalind J. Allen, Tine Curk, James Farrell, Gina McAllister, Kate Templeton, Till T. Bachmann

**Affiliations:** † Infection Medicine, Edinburgh Medical School: Biomedical Sciences, University of Edinburgh, Chancellor’s Building, 49 Little France Crescent, Edinburgh, EH16 4SB, United Kingdom; ‡ School of Physics and Astronomy, University of Edinburgh, Edinburgh, EH9 3FD, United Kingdom; § Department of Materials Science and Engineering, Northwestern University, Evanston, Illinois 60208, United States; ∥ Institute of Physics, Chinese Academy of Sciences, Beijing, 100190, China; ⊥ School of Physical Sciences, University of Chinese Academy of Sciences, Beijing, 100049, China; # Department of Laboratory Medicine, Royal Infirmary of Edinburgh, Edinburgh, EH16 4SA, United Kingdom

**Keywords:** SARS-CoV-2, electrochemical biosensor, point-of-care diagnostics, multi-valent binding, electrochemical impedance spectroscopy.

## Abstract

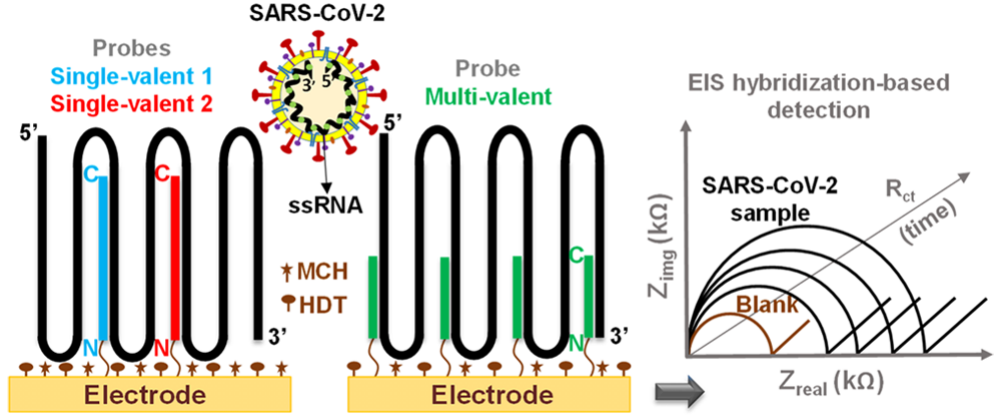

We present the development of electrochemical impedance spectroscopy (EIS)-based biosensors for sensitive detection of SARS-CoV-2 RNA using multi-valent binding. By increasing the number of probe–target binding events per target molecule, multi-valent binding is a viable strategy for improving the biosensor performance. As EIS can provide sensitive and label-free measurements of nucleic acid targets during probe–target hybridization, we used multi-valent binding to build EIS biosensors for targeting SARS-CoV-2 RNA. For developing the biosensor, we explored two different approaches including probe combinations that individually bind in a single-valent fashion and the probes that bind in a multi-valent manner on their own. While we found excellent biosensor performance using probe combinations, we also discovered unexpected signal suppression. We explained the signal suppression theoretically using inter- and intra-probe hybridizations which confirmed our experimental findings. With our best probe combination, we achieved a LOD of 182 copies/μL (303 aM) of SARS-CoV-2 RNA and used these for successful evaluation of patient samples for COVID-19 diagnostics. We were also able to show the concept of multi-valent binding with shorter probes in the second approach. Here, a 13-nt-long probe has shown the best performance during SARS-CoV-2 RNA binding. Therefore, multi-valent binding approaches using EIS have high utility for direct detection of nucleic acid targets and for point-of-care diagnostics.

In 2020, the devastating pandemic of Coronavirus disease 2019 (COVID-19) emerged as a result of rapid human-to-human transmission of the severe acute respiratory syndrome 2 virus (SARS-CoV-2). The success of the response to the pandemic was critically dependent on diagnostics and created a huge global demand for suitable COVID-19 tests to help with rapid detection and isolation of positive cases. Presently, most countries rely on serological and viral nucleic acid tests for COVID-19 diagnostics.^1−3^ Several nucleic acid based methods such as real-time reverse transcription quantitative polymerase chain reaction (RT-qPCR),^4−6^ clustered regularly interspaced short palindromic repeats (CRISPR),^7,8^ and isothermal amplification^9,10^ have been reported for SARS-CoV-2 detection. Among them, RT-qPCR is used globally as a gold standard for detecting viral RNA. Nonetheless, RT-qPCR has some shortcomings, including the requirement for costly instruments, reagents, and trained personnel, transportation of samples to reference laboratories, and a longer sample-to-result time.^11,12^ Therefore, rapid, accurate, and easy-to-implement methods for SARS-CoV-2 RNA detection are still an unmet need. Previous studies addressed direct detection of SARS-CoV-2 but require assay procedures which limit their suitability for point-of-care testing.^13−15^

Point-of-care test compatible biosensors, especially those using electrochemical transducers, provide a good alternative to PCR analysis owing to their on-site detection capabilities, low cost, easy operation, and scalability for mass production.^16,17^ Due to their simplicity and ease of miniaturization, electrochemical biosensors are especially advantageous in clinical diagnostics and point-of-care testing (POCT).^18^ In particular, electrochemical impedance spectroscopy (EIS) has previously been used to build fast and sensitive tests for nucleic acid assays.^19−21^ EIS methods allow for single-step and label-free measurements of the targets during nucleic acid hybridization events utilizing simple hand-held instrumentations and readout.^22,23^ This could help with the development of a rapid and easy screening technology for COVID-19. EIS biosensors for nucleic acid testing generally use sequence-specific single-strand nucleic acid probes which are immobilized on the electrode surfaces. Conventionally, these probes are designed to be complementary to only one region of the target molecule. While the individual probe binds strongly, the overall target capture is dependent on only one binding event.^12^ In contrast, multi-valent binding, in which several regions of the target nucleic acid hybridize simultaneously to the probe (or probes), provides an alternative approach with potential advantages. By increasing the number of probe–target binding sites, multi-valent binding could enhance the sensitivity of the biosensor. Our recent computational modeling study suggested that the design of short oligonucleotide probes for multi-valent binding to a nucleic acid target could lead to high sensitivity and selectivity, especially for long targets and in the case where probe design took account of both target and nontarget sequences.^24^ In the present study, we developed EIS biosensors for multi-valent targeting SARS-CoV-2 RNA. We compared two approaches to build the biosensor: (1) combinations of probes that each bind in a single-valent manner and (2) probes that bind multi-valently on their own. This study suggests that multi-valent binding is a highly promising approach for direct detection of nucleic acids in the development of molecular diagnostics at point-of-care.

## Experimental Section

### Reagents, Probes, and Targets

Tris(2-carboxyethyl)phosphine hydrochloride (TCEP), sulfuric acid (H_2_SO_4_), dimethyl sulfoxide (DMSO), dimethylformamide (DMF), sodium chloride (NaCl), monosodium phosphate (NaH_2_PO_4_), disodium phosphate (Na_2_HPO_4_), potassium ferricyanide {K_3_Fe(CN)_6_}, and potassium ferrocyanide {K_4_Fe(CN)_6_} were purchased from Sigma-Aldrich (Gillingham, UK). 6-Mercapto-1-hexanol (MCH) and 1,6-hexanedithiol (HDT) were procured from ProChimia Surfaces (Gdynia, Poland). All of the other reagents were of analytical grade and used without any further purification. All aqueous solutions were made with deionized water (resistivity >18 MΩ cm) from a Millipore Milli-Q water purification system (Bedford, MA, USA). Peptide nucleic acid (PNA) single-stranded probes were ordered via Cambridge Research Biochemicals (Cleveland, UK) and obtained from Panagene (Daejeon, South Korea). Probes (>95% HPLC purified) were synthesized with an 11-mercapto-1-undecanol linker on the N-end of the PNA (equivalent to 5′-end of DNA) for specific attachment onto the gold surfaces via self-assembly. Stock solutions of PNA probe were made with 50% (v/v) dimethylformamide (DMF) aqueous solution and used further for sensing layer formation. Exact size-matched DNA (T-RdRp1_,_ T-RdRp2, T-RdRp3, T-N1, T-MV1, T-MV2, and T-MV3) and MV3 RNA targets were the reverse complementary sequences of their respective probes. DNA and RNA target sequences were bought from Metabion (Martinsried, Germany) and used after preparing stock solutions by dissolving lyophilized targets into nuclease-free deionized (DI) water. The stock solutions of PNA probe and DNA target were both kept at −20 °C when not in use. Details of the sequence and structure of PNA probes and DNA or RNA targets are given in Table S1. Buffer for diluting SARS-CoV-2 RNA after bench-extraction was purchased from Takara Bio Europe (Saint-Germain-en-Laye, France) and preserved at −20 °C during storage. Remel MicroTest M4RT viral transport media (VTM) was purchased from Thermo Fisher Scientific (Waltham, MA, USA).

### Probe Design

Single-valent probe sequences were designed after selecting three target regions from the RNA-dependent RNA polymerase (RdRp) gene^4^ and one target region of the nucleocapsid protein (N) gene^6^ of the SARS-CoV-2 genome. The P-RdRp1, P-RdRp2, and P-RdRp3 sequences are specific for the target regions at 15431–15452 bp, 15505–15530 bp, and 15470–15494 bp, respectively, whereas the P-N1 sequence is specific for the 28287–28306 bp region of the SARS-CoV-2 genome (see Scheme S1). For the multi-valent probe design, please see the Supplementary Experimental Section in Supporting Information. PNA probes were modified with a spacer comprising three ethylene glycol units (abbreviated as AEEEA) and a terminal thiol group at the N-end. Details on theoretical calculation for intra- and inter-probe interactions, statistics for data analysis, and the preparation of SARS-CoV-2 RNA from cell culture or patient samples can be found in Supplementary Experimental Section.

### Electrode Preparation

**Figure 1:**
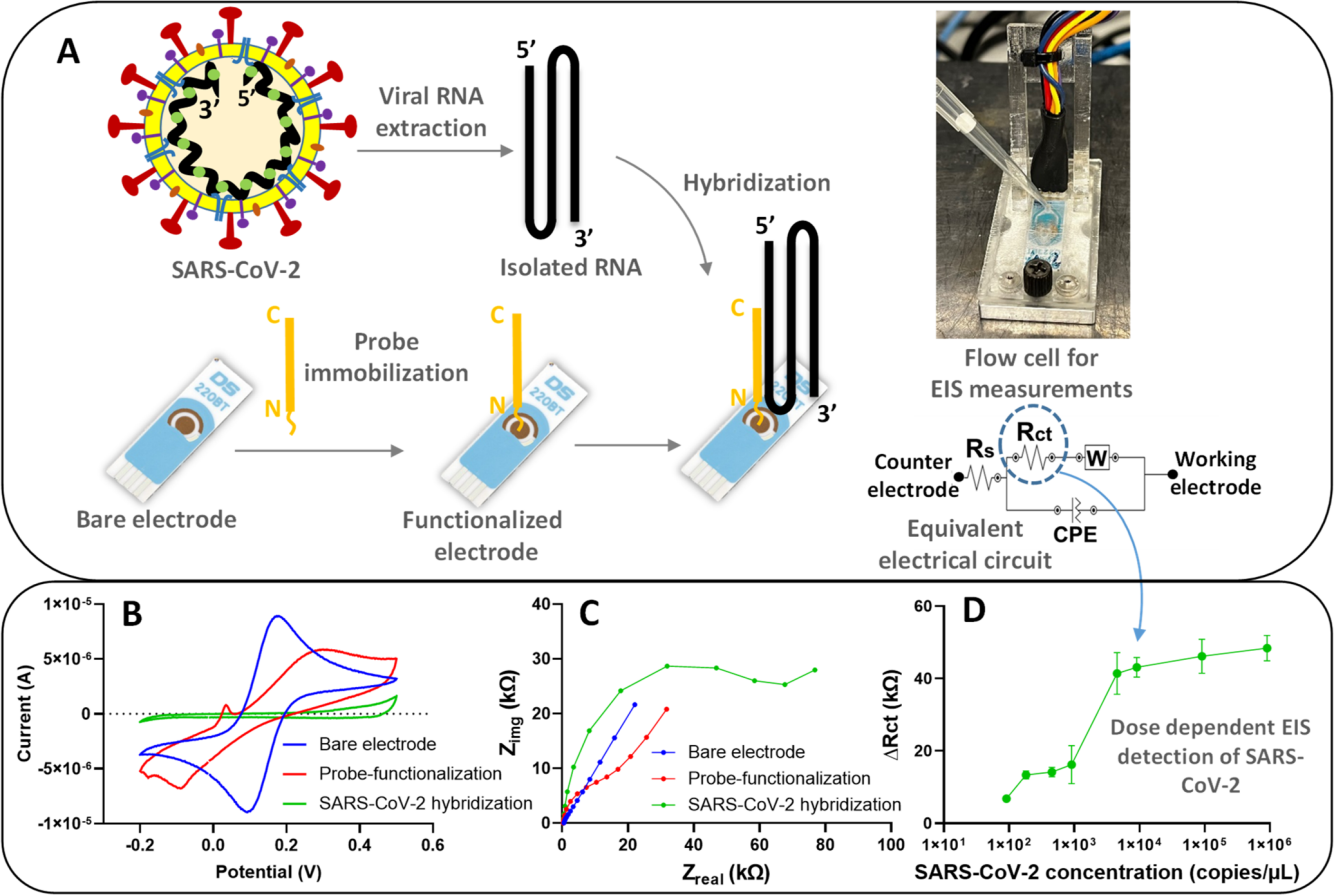
Electrode preparation, characterization, and SARS-CoV-2 detection: (A) process showing electrode preparation for SARS-CoV-2 RNA hybridization, flow cell for electrochemical measurements, and electrical circuit for electrochemical impedance spectroscopy (EIS) Nyquist plot fitting, (B) cyclic voltammetry, (C) EIS Nyqusit plot characterizations at each surface modification of electrode, and (D) dose dependent detection of SARS-CoV-2 RNA (9.09 × 10^1^ – 9.09 × 10^5^ copies/μL) after electrical circuit fitting of Nyquist plots and interpretation on charge transfer resistance (*R*_ct_). *R*_s_, W, and CPE represent solution resistance, Warburg element, and constant phase element, respectively.

Screen-printed gold electrodes (DRP-C223BT, DropSens) were functionalized with PNA probes as per the protocol used in our earlier study.^25^ In brief, following electrochemical cleaning using 100 mM sulfuric acid solution and cyclic voltammetry technique (0 to 1.6 V potential range, 100 mV/s scan rate, 10 cycles), the PNA probe molecules were immobilized onto the gold working electrodes by exposing the cleaned electrodes with a mixed solution containing specific concentrations of the probe (thiol-modified PNA probe + 100 μM MCH + 200 μM HDT + 5 mM TCEP in 50% DMSO solution) for 16 h followed by blocking with 1 mM MCH solution for 2 h. Finally, the probe-functionalized electrodes were serially rinsed with 50% (v/v) DMSO aqueous solution and DI water and then used for subsequent impedance measurements (see Figure 1).

### Electrochemical Impedance Spectroscopy (EIS) Measurements

All electrochemical measurements including EIS were conducted using an Autolab PGSTAT128N potentiostat/galvanostat system (Metrohm Autolab, Utrecht, Netherlands). EIS measurements were recorded in the frequency range 0.3 Hz to 100 kHz with a signal amplitude of 10 mV rms at the measured open circuit potential. Nyquist plots for each measurement were used to fit the data in an equivalent Randles’ circuit and to calculate the charge transfer resistance (*R*_ct_) values using the NOVA 2.1 software. The Randles’ equivalent circuit was designed with a constant phase element (as nonideal capacitance) in place of the double layer capacitance (*C*_dl_) and the corresponding changes in the *R*_ct_ values were considered in the Faradaic EIS measurements. EIS measurements were performed pre and post hybridization with a 35 min sample incubation using probe-functionalized electrodes, and the increase in *R*_ct_ values (Δ*R*_ct_) from pre (baseline measurement) to post (sample measurement) hybridization was considered during the plotting of impedance data. All EIS studies were performed in 10 mM sodium phosphate buffer, pH 7 with 20 mM sodium chloride and 0.2 mM potassium ferri/ferrocyanide redox mediator (EIS measurement buffer), while the cyclic voltammetric characterization of electrodes was performed with 10 times concentrated EIS measurement buffer (see Figure 1).

## Results

### Design of Single-Valent Probes

**Figure 2:**
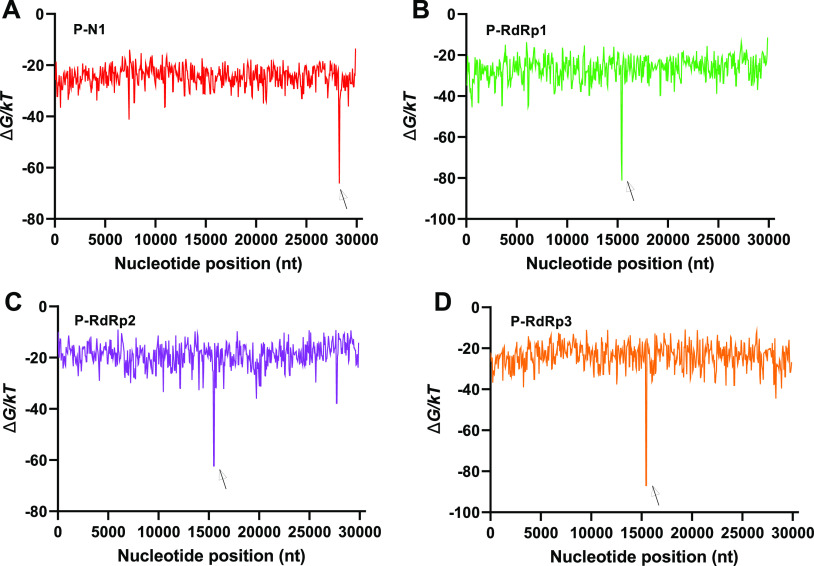
Theoretical free energy (Δ*G*) of SARS-CoV-2 target (nc045512) for binding with (A) P-N1, (B) P-RdRp1, (C) P-RdRp2, and (D) P-RdRp3 probes. The arrows indicate the respective binding regions. Predicted free energy of binding Δ*G*, of the SARS-CoV-2 genome to selected probes, resolved by position along the genome. To make these plots, the SARS-CoV-2 was split into 100 nt sections. For each section of the genome, the free energy of binding to the probe Δ*G* was calculated using NuPack,^27^ with parameters for RNA–RNA interactions at 1 M salt (to screen out electrostatic interactions) and 20 °C,^28^ and without considering intra-genome binding to model PNA–DNA interactions, as described in the Supplementary Experimental Section.

As a member of the coronavirus family, SARS-CoV-2 possesses single-stranded positive-sense RNA (+ssRNA) which is ∼3 kb in length.^26^ As shown in Scheme S1, the SARS-CoV-2 genome comprises the 5′ untranslated region (UTR), replicase complex (ORF1ab), spike surface glycoprotein gene (S gene), small envelope gene (E gene), matrix gene (M gene), nucleocapsid gene (N gene), 3′ UTR, and several nonstructural open reading frames. We designed four probes (P-N1, P-RdRp1, P-RdRp2, and P-RdRp3) to bind in a single-valent manner, i.e., for one binding site in the SARS-CoV-2 genome each. We verified the binding of SARS-CoV-2 target with the respective probes by calculating probe–genome interaction free energy using NuPack (Figure 2). Each probe showed a strong binding signal at the respective complementary target region.

### Performance of Single-Valent Probes with Size-Matched DNA Target

The performance of each single-valent probe was investigated at the same probe concentration (9 μM) using EIS. The relative strength of the measured EIS signals (Δ*R*_ct_) was, in order of intensity: P-N1 > P-RdRp1 > P-RdRp2 > P-RdRp3 (Figure S6). Probe P-RdRp2 had a low EIS response, presumably due to the formation of several secondary structures. No further work was conducted subsequently with the P-RdRp3 probe because of its poor response (*p* = 0.96 w.r.t blank measurements). We found 7 and 4 self-annealing sites for P-RdRp2 and P-RdRp3, respectively, while both the probes showed one hairpin loop formation structure.

For our planned multi-valent binding of SARS-CoV-2 RNA, we first considered the optimal single-valent probe concentrations (Figure S1). Next, we combined two or three of the single-valent probes together, to achieve multi-valent binding of the target. Please see Supplementary Results Section for the details on effect of probe concentration, probe combination, signal suppression, hybridization temperature, and our theoretical calculation to explain response suppression during the probe combinations.

### SARS-CoV-2 RNA Detection with Single-Valent Probes

**Figure 3:**
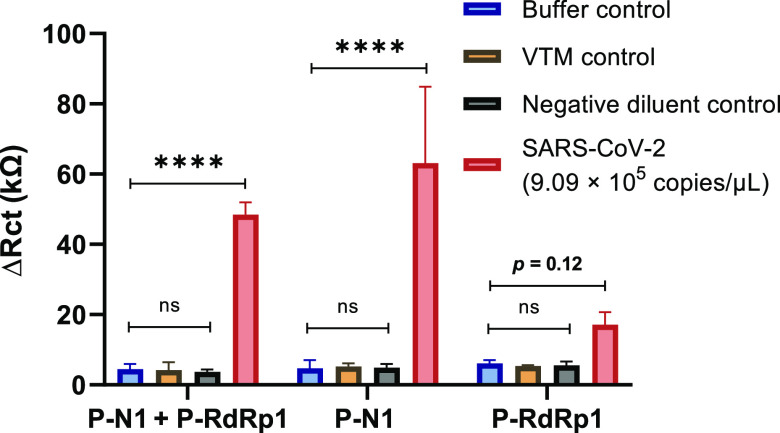
Direct detection of SARS-CoV-2 RNA with single-valent probes: EIS signals (Δ*R*_ct_) of electrodes functionalized with P-N1 and P-RdRp1 either alone or in combination at 3 μM each after 35 min incubation at 50 °C with buffer, viral transport media (VTM) control, negative diluent control, or SARS-CoV-2 RNA (9.09 × 10^5^ copies/μL). Data represent the mean ± SD; *n* = 3.

To test the utility of our probe combinations for direct detection of SARS-CoV-2 RNA, we investigated the performance of the single-valent probes with a long, native RNA target from cell culture, consisting of the SARS-CoV-2 RNA genome. To this end, we performed EIS measurements for the P-N1 + P-RdRp1 combination at equimolar concentration (3 μM each), and for the individual P-N1 (3 μM) and P-RdRp1 (3 μM) with SARS-CoV-2 RNA (9.09 × 10^5^ copies/μL) at 50 °C (Figure 3). As negative controls, we recorded the signals after incubation with reagents used in the sample preparation of the SARS-CoV-2 RNA and the EIS buffer. We found a strong, significant enhancement in the EIS signal for the P-N1 + P-RdRp1 combination and the P-N1 alone (*p* < 0.0001 in both cases) upon addition of the SARS-CoV-2 RNA target. The P-RdRp1 showed a less significant signal increase (*p* = 0.12) upon target addition. Importantly, we did not observe any significant signal increase for the negative controls.

### Dose Dependence of SARS-CoV-2 RNA Detection and COVID-19 Patient Sample Analysis

To investigate whether our biosensor could detect SARS-CoV-2 RNA at clinically relevant concentrations, we studied its response to a dilution series of RNA derived from the same SARS-CoV-2 sample and the P-N1 + P-RdRp1 combination (3 μM each) at 50 °C (see Figure S7 for the overlay of Nyquist and Bode plots). The dose response curve (Figure 1D or S8) for the EIS studies with SARS-Cov-2 RNA concentrations showed an EIS signal (Δ*R*_ct_) that correlated strongly with the target concentration. We obtained a Limit of Detection (LOD) of 182 copies/μL (equivalent to 303 aM) and Limit of Quantitation (LOQ) of 4550 copies/μL (equivalent to 7.58 fM) based on the blank measurements^29^ (mean value 4.45 kΩ, standard deviation ±1.52 kΩ, and *n* = 3).

**Figure 4:**
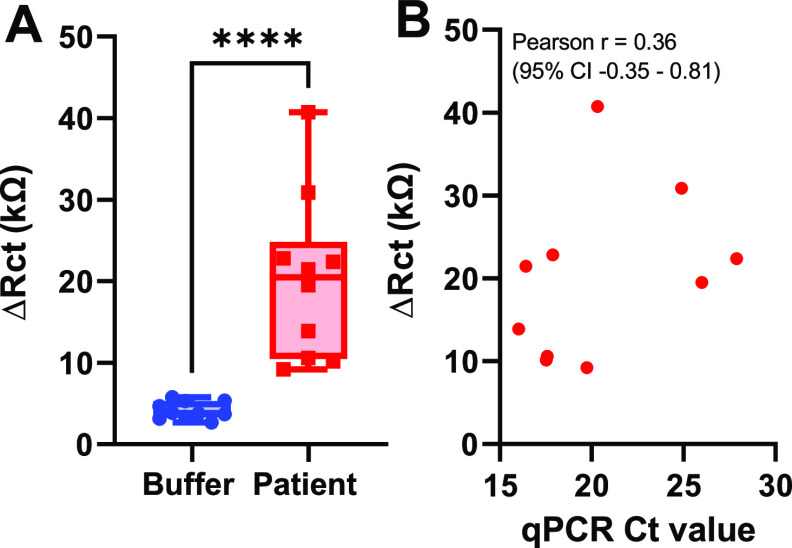
COVID-19 patient sample analysis: (A) EIS signals (Δ*R*_ct_) of electrodes functionalized with the combination of P-N1 and P-RdRp1 (3 μM each) after 35 min incubation at 50 °C with either COVID-19 positive samples (1:2.5 dilution with measurement buffer and deionized water) or measurement buffer control. Data represent the mean ± SD; *n* = 10. (B) Pearson correlation showing the relationship between the sensor signal (Δ*R*_ct_) and gold standard qPCR method (*C*_t_ value).

For further investigation with real patient samples, SARS-CoV-2 RNA from COVID-19 positive samples were analyzed, and the results (Figure 4) demonstrate a significant increase (*p* < 0.0001), as compared to background (buffer control), and a decent correlation (Pearson *r* = 0.36) with the gold standard qPCR method.

### Design of Multi-Valent Probes

**Figure 5:**
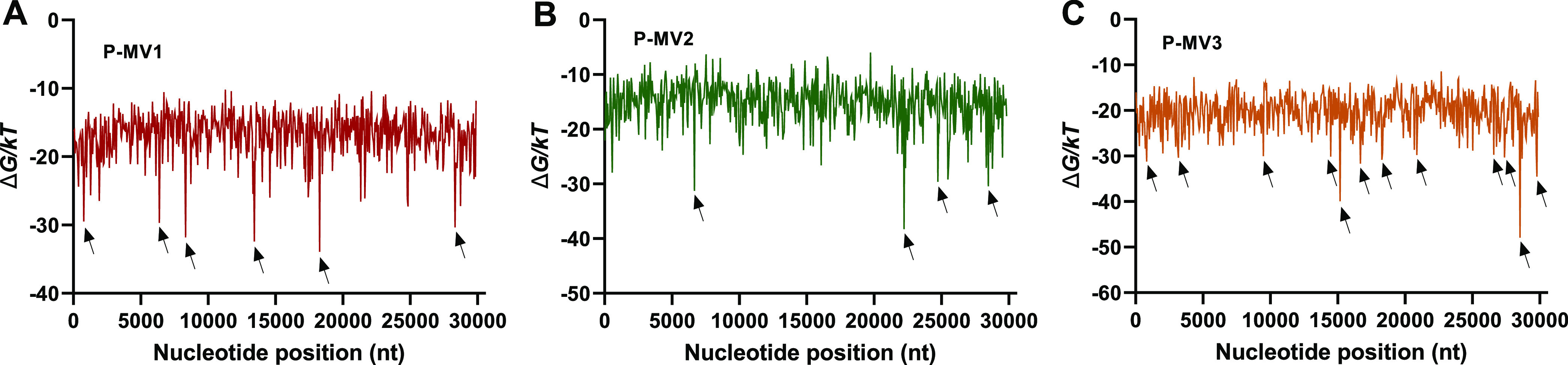
Binding free energy (Δ*G*) of SARS-CoV-2 target (nc045512) for binding with (A) P-MV1, (B) P-MV2, and (C) P-MV3 probes, predicted using NuPack (see Supplementary Experimental Section). The arrows indicate the binding regions.

We designed three shorter probes (8, 10, and 13 nt in length) to bind multi-valently to the SARS-CoV-2 RNA, i.e., to have multiple binding sites on the target RNA (Figure 5). The probe design approach also ensured that our multi-valent probes would bind specifically to SARS-CoV-2 rather to other coronavirus genomes (see Supplementary Experimental Section). By using short probes we hoped to avoid within-probe secondary structure formation. By designing the probes to bind multi-valently, we hoped to achieve the advantages of multi-valent binding, without encountering the problems with probe–probe interaction that we observed during the co-immobilization of single-valent probes.

### SARS-CoV-2 RNA Detection with Multi-Valent Probes

**Figure 6:**
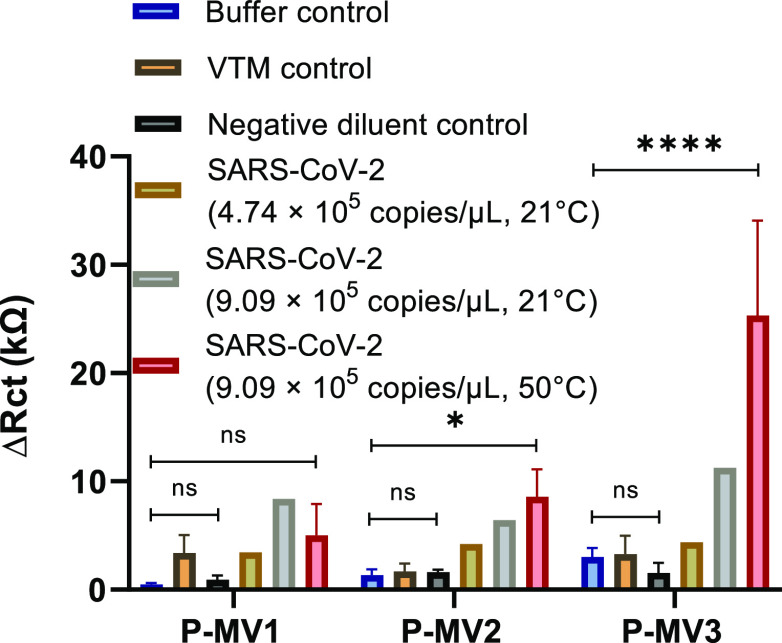
Direct detection of SARS-CoV-2 RNA with multi-valent probes: EIS signals (Δ*R*_ct_) of electrodes functionalized with 6 μM solutions of P-MV1, P-MV2, and P-MV3, after 35 min sample incubation with buffer, viral transport media (VTM), negative diluent control, or SARS-CoV-2 RNA samples of 4.74 × 10^5^ copies/μL at 21 °C, and 9.09 × 10^5^ copies/μL at 21 and 50 °C.

We analyzed solutions of SARS-CoV-2 RNA at two different concentrations (9.09 × 10^5^ copies/μL and 4.74 × 10^5^ copies/μL) at room temperature (21 °C) with the P-MV1, P-MV2, and P-MV3 probes at 6 μM probe concentration. We also studied the SARS-CoV-2 solution with 9.09 × 10^5^ copies/μL concentration at 50 °C. All three multi-valent probes (P-MV1, P-MV2, and P-MV3) showed higher signals for the SARS-CoV-2 sample with 9.09 × 10^5^ copies/μL concentration as compared to the negative controls both at room temperature and 50 °C (Figure 6). The P-MV2 and P-MV3 probes displayed a further increase in the EIS signals (Δ*R*_ct_) at 50 °C as compared to room temperature.

### Multi-Valent Binding Analysis with P-MV3 Probe

To check the multi-valent binding of the target, we took our best performing multi-valent probe P-MV3 and did dose dependence studies for the size-matched RNA oligo (single biding site) and the full-length SARS-CoV-2 RNA (multiple binding sites). We observed a lower equilibrium binding constant (*K*_D_ = 19.64 fM) for the SARS-CoV-2 target (multiple binding sites in target) as compared to the size matched RNA oligo target with only one binding site (*K*_D_ = 94.01 nM) (Figure S10). We anticipate that the lower *K*_D_ value resulted from the multi-valent binding of the target with P-MV3.

### Comparison of Single-Valent Probe Combination with Multi-Valent Probes for SARS-CoV-2 RNA Detection

**Figure 7:**
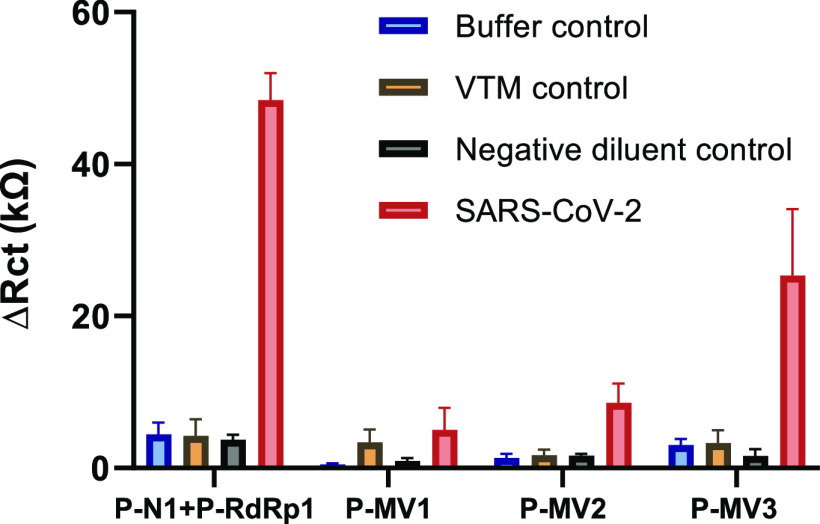
Comparison of direct detection of SARS-CoV-2 RNA by a single-valent probe combination with multi-valent probes: EIS signals (Δ*R*_ct_) of electrodes functionalized with either the combination of P-N1 and P-RdRp1 (3 μM each) or P-MV1, P-MV2, or P-MV3 (6 μM each) after 35 min sample incubation at 50 °C with buffer, viral transport media (VTM), negative diluent control, or SARS-CoV-2 RNA sample (9.09 × 10^5^ copies/μL). Data represent the mean ± SD; *n* = 3.

To compare the two ways of achieving multivalency, combinations of single-valent probes and the use of individual multi-valent probes, we studied the EIS responses (Δ*R*_ct_) of the P-N1 + P-RdRp1 combination (3 μM each) and the P-MV1, P-MV2, and P-MV3 multi-valent probes (6 μM). We used a single SARS-CoV-2 RNA sample (9.09 × 10^5^ copies/μL) to ensure the same conditions for all probes and incubated at 50 °C for 35 min. Both the P-N1 + P-RdRp1 probe combination and the P-MV3 multi-valent probe produced strong signals, although the signal from the other multi-valent probes was less strong (Figure 7). We used a microRNA-specific control probe (P-miR122), which showed a lower response than the P-N1 + P-RdRp1 probe combination and the P-MV3 probe (Figure S9). Therefore, further investigation of the sensitivity and selectivity properties of both the dual combination of single-valent probes and of the multi-valent P-MV3 would be useful (e.g., at different probe concentrations and different concentrations of the target). However, the measurements performed in this study suggest that the combination of the two single-valent probes P-N1 + P-RdRp1 has higher sensitivity than the designed multi-valent probes, despite the presence of signal suppression due to probe–probe interactions.

## Discussion

As the COVID-19 pandemic has shown, it is highly desirable to detect SARS-CoV-2 RNA at point-of-care. Here, we aimed to develop electrochemical biosensors for COVID-19 POCT by functionalizing commercially available screen-printed electrodes with SARS-CoV-2 RNA specific PNA probes following two strategies: (i) combinations of probes with a single target binding region each and (ii) individual probes with multiple target binding regions each.^24^ Our most important finding was that a combination of single-valent probes can perform well for direct detection of the SARS-CoV-2 RNA target, with a clinically relevant detection limit. We achieved a detection limit of 182 copies/μL (303 aM) for SARS-CoV-2 RNA. This is well within the relevant clinical range of SARS-CoV-2 RNA as the viral load is often between 10^1^ and 10^5^ copies/μL in throat swaps and sputum samples on days 1 to 8 after onset of the disease.^11,30,31^ As a comparison, typical detection limits for SARS-CoV-2 RNA RT-qPCR assays are in the range of 0.45–7.8 copies/μL.^4,5,32^ For POCT detection of SARS-CoV-2 DNA/RNA, a wide range of different methods have been proposed.^12,33−36^ Among studies that report detection limits for the whole SARS-CoV-2 RNA genome, some have obtained more sensitive detection, but at the cost of greater methodological complexity. For example, Zhao et al. obtained a LOD of 0.2 copies/μL for SARS-CoV-2 RNA from the clinical specimens using calixarene functionalized graphene oxide combined with a sandwich-type assay and differential pulse voltammetry (DPV),^12^ while Kong et al. obtained a LOD of 0.03 copies/μL for SARS-CoV-2 nucleic acid (cDNA and *in vitro* transcribed RNA) detection using a Y-shaped DNA dual probe-functionalized graphene-field effect transistor to simultaneously target the ORF1ab and N genes.^35^

This strong performance of our biosensor occurred despite the fact that we found combinations of single-valent probes to be prone to signal suppression. Our study suggests that both secondary structure formation within probes and probe–probe hybridization can significantly suppress target binding. This conclusion emerges from the fact that we could account quantitatively for our response suppression data using a theoretical analysis based on the thermodynamics of intra- and inter-probe binding, that assumed only unhybridized probe monomers could contribute to target binding. These observations complement those of a previous study by Gao et al., who showed that the presence of secondary structures in probes, as well as in targets, can adversely affect DNA–DNA hybridization kinetics both in solution and on surfaces.^37^ Indeed, since target-probe binding is far more thermodynamically favorable than inter- or intra-probe binding, our observation points to the relevance of kinetic effects in probe–target binding.

For multi-valent probes, we did not expect the same signal suppression issue, since here one does not need to use probe mixtures, and the multi-valent probes were also shorter, reducing intra-probe self-hybridization potential. Our previous computational study has shown that multi-valent probes can lead to higher sensitivity and specificity for detection of long DNA targets.^24^ Multiple binding sites produce strong overall binding (even if individual binding sites are weak), leading to high sensitivity. In this study, we indeed obtained good sensitivity for the multi-valent P-MV3, although the dual combination of single-valent probes showed somewhat higher sensitivity. Perhaps the length of the SARS-CoV-2 RNA (∼3 kb) was too short to fully realize the benefits of multi-valent probe design.

Our work shows a simple, low-cost, and easy-to-implement EIS-based method for detection of SARS-CoV-2 RNA at point-of-care that can give a LOD within the clinical range. To our knowledge, our study is the first to use EIS for direct detection of SARS-CoV-2 RNA. Most other reported EIS-based techniques for SARS-CoV-2 detection have targeted the spike protein or have been immunoassay-based,^19,38−40^ although EIS-based detection of the whole SARS-CoV-2 particle has been reported.^41^

The measurements performed in this study suggest that multi-valent binding, combined with EIS, can be a promising approach for direct detection of SARS-CoV-2 RNA. Further investigation of the properties of the multi-valent probes would be useful (e.g., at different probe concentrations and different concentrations of the target). As demonstrated by various theoretical and experimental studies,^24,42−44^ multi-valent probes can have superselective targeting properties, which should aid in achieving better specificity in detecting SARS-CoV-2 RNA from samples containing other similar viruses. Therefore, specificity studies of the designed multi-valent probes for the SARS-CoV-2 target, and a comparison of specificity performance with the single-valent probe combination, would be intriguing and relevant in future research. In particular, we plan to investigate the specificity of the designed probes in the presence of other common cold corona viruses, such as HCoV-OC43, HCoV-HKU1, HCoV-229E, and HCoV-NL63.

## Conclusions

We have demonstrated direct, amplification free detection of SARS-CoV-2 RNA with clinically relevant sensitivity using an EIS biosensor and a multi-valent binding approach. Two approaches, single-valent probe combination and multi-valent probes, were found feasible. We further found that multiple probe combinations can lead to unexpected signal suppression and provided a theoretical model to explain these. In summary, multi-valent target binding is highly promising for direct detection of SARS-CoV-2 RNA and likely offers significant opportunities for molecular diagnostics of other diseases at point-of-care.

## Supporting Information Available

Supplementary experimental section, supplementary results section, nomenclature and sequence of probes and targets, SARS-CoV-2 structure and genome organization with target regions, single-valent probe-to-probe comparison, dose dependence studies of SARS-CoV-2 RNA using the combination of P-N1 and P-RdRp1, overlay of Nyquist and Bode plots, comparison of specific probes with negative control probe, dose dependent target detection for analyzing multivalency of P-MV3 (PDF)
