# Supplementary material for: Amplification Free Detection of SARS-CoV-2 Using Multi-Valent Binding
Source: ACS Sens. 2022 Dec 9;7(12):3692–9. doi: 10.1021/acssensors.2c01340 (PMC9743695; doi:10.1021/acssensors.2c01340)
Supplement: Supplementary file 1 — se2c01340_si_001.pdf [file se2c01340_si_001.pdf]

Supporting Information for Publication

Amplification Free Detection of SARS-CoV-2 Using Multi-Valent Binding

Appan Roychoudhury[1], Rosalind J. Allen[2], Tine Curk[3], James Farrell[4,5], Gina McAllister[6], Kate Templeton[6], Till T. Bachmann[1*]

[1]Infection Medicine, Edinburgh Medical School: Biomedical Sciences, University of Edinburgh, Chancellor's Building, 49 Little France Crescent, Edinburgh, EH16 4SB, UK

[2]School of Physics and Astronomy, University of Edinburgh, Edinburgh, EH9 3FD, UK

[3]Department of Materials Science and Engineering, Northwestern University, Evanston, IL 60208, USA

[4]Institute of Physics, Chinese Academy of Sciences, Beijing, 100190, China

[5]School of Physical Sciences, University of Chinese Academy of Sciences, Beijing, 100049, China

[6]Department of Laboratory Medicine, Royal Infirmary of Edinburgh, Edinburgh, EH16 4SA, UK

*Corresponding author E-mail: till.bachmann@ed.ac.uk

Table of contents:

	Description	Page no.
Supplementary Experimental Section	Theoretical calculations for intra- and inter-probe interactions, multi-valent probe design, preparation of full-length SARS-CoV-2 RNA from cell culture and COVID-19 patient samples, statistics	3-4
Supplementary Results Section	Optimal single-valent probe concentrations, co-immobilization causes response suppression, inter- and intra-probe hybridization can explain response suppression, temperature dependence of single-valent probes binding to size-matched DNA targets, performance of multi-valent probes with size-matched DNA targets	4-9
Table S1	Nomenclature and sequence of PNA probes and DNA targets used in present study	10
Scheme S1	SARS-CoV-2 structure and genome organization indicating structural features and target regions. RNA-dependent RNA polymerase (RdRp) and nucleocapsid protein (N) gene regions were used for designing single-valent probes	11
Figure S6	Single-valent probe-to-probe comparison	11
Figure S7	Overlay of Nyquist and Bode plots during dose dependence studies of SARS-CoV-2 RNA using the combination of P-N1 and P-RdRp1 probes	12
Figure S8	Dose dependent direct detection of SARS-CoV-2 RNA using the combination of P-N1 and P-RdRp1 probes	12
Figure S9	Comparison of specific probe or probe combination with a negative control probe for SARS-CoV-2 RNA detection	13
Figure S10	Dose dependent target detection for analyzing multi-valency of P-MV3 probe	13
References		13

Supplementary Experimental Section

Theoretical Calculations for Intra- and Inter-probe Interactions. For the 3 single-valent probes (P-N1, P-RdRp1 and P-RdRp2), we used NuPack[1] to predict the free energy change upon formation of hybridized monomers, dimers and trimers, and of dimer and trimer complexes for all probe combinations. Because NuPack parameters are not available for PNA, we used parameters for RNA, and we performed the calculations at high salt concentration (1 M), to remove any contribution arising from electrostatic interactions. We then used an in-house Mathematica script to calculate the equilibrium composition, in terms of unhybridized and hybridized probe monomers, dimer and trimer complexes, for a given probe concentration. The appropriate probe concentration to use in this calculation is unknown, since we do not know the probe density on the electrode surface. However we observed that the predicted response suppression factor was independent of probe concentration for high enough probe concentration. Therefore, we used a concentration of 0.05 M for each probe, in all our calculations. To predict the response suppression factor, we assumed that only unhybridized monomers contribute to the response. Therefore, for a given combination of probes, we calculated the unhybridized monomer concentrations for each probe in isolation (at concentration 0.05 M), and for the probe mixture (with each component at concentration 0.05 M). We then obtained the theoretical response suppression factor as the sum of the unhybridized monomer concentrations in isolation, divided by the unhybridized monomer concentration for the mixture.

Multi-valent Probe Design. Three multi-valent probe sequences of length 8 nt (P-MV1), 10 nt (P-MV2) and 13 nt (P-MV3) were designed for selective binding of the SARS-CoV-2 RNA. We used a two-stage approach to design these probes.

In the first stage, we assessed the suitability of all possible nucleotide sequences of the desired length for binding to both the SARS-CoV-2 genome (nc045512) and to a common cold coronavirus (nc002645), using a score function approach.[2] We used the score function $S = \log[\sum_{a=1}^{l} e^{2a} n_a]$, where l denotes the probe length and n_a the number of continuous matches of length a between the probe and the genome. This score function takes into account both the binding strength and the multiplicity of binding sites.[2] To promote selectivity of binding, we assigned a score $\Delta S = S_{\mathrm{nc045512}} - S_{\mathrm{nc002645}}$ to every possible probe based on the difference in its multi-valent binding scores for the SARS-CoV-2 and the common cold coronavirus genomes.

In the second stage, we further assessed the highest-scoring candidate probe sequences, using NuPack (http://www.nupack.org) to calculate the free energy of binding to both the SARS-CoV-2 and the common cold coronavirus genomes.[1] Since parameters are not available for PNA, we approximated the PNA-RNA interaction using parameters for RNA-RNA interactions at 1 M salt (to screen out electrostatic interactions) and 20°C.[3] To design the 8 nt and the 10 nt probes we also modified the NuPack code to remove the contributions of intra-genome RNA-RNA interactions, since these are expected to be dominated by probe-genome PNA-RNA interactions.

To favor probes that bind multi-valently, we split the genomes into 5000 nt sections. For the 8 nt and 10 nt probes, we further split each 5000 nt section into segments of 100 nt and evaluated the binding free energy of the candidate probe with each of the 100 nt segments. For the 13 nt probe, each 5000 nt section was split into smaller, overlapping, segments of 20 nt, with a 10 nt step size. Small segments were used to reduce the contribution of intra-genome RNA-RNA interactions to the calculation. In all cases, we performed an exponential average over the segments and averaged this over the 5000 nt sections, to obtain an estimated free energy of binding of the candidate probe to the genome: $\Delta G = -kT\langle log\langle e^{-\Delta G_{ij}}\rangle_j\rangle_i$, where the index j denotes a 100 nt segment, i denotes a 5000 nt section, and $\langle x\rangle_i$ denotes an average of x over all i, and ΔG_{ij} denotes the SantaLucia free energy between the probe and the j segment in i-th section. This quantity was calculated for the SARS-CoV-2 and the common cold coronavirus genomes. For the probes of length 10 nt and 13 nt, this procedure was used to evaluate the 1000 top-scoring candidate probes from stage 1, and the probe with the most negative value of $\Delta G_{nc045512} - \Delta G_{nc0026645}$ (i.e. the difference in predicted binding free energy between the SARS-CoV-2 and common cold genomes) was chosen (**Table S1**). For probes of length 8 nt, we evaluated the 10,000 top-scoring probes from stage 1.

Preparation of Full-length SARS-CoV-2 RNA from Cell Culture and COVID-19 Patient Samples. A clinical sample collected from a patient for SARS-CoV-2 testing at the Royal Infirmary of Edinburgh was used to culture SARS-CoV-2 virus in a Vero cell line. The cell culture supernatant was inactivated by mixing 1 : 1 with lysis buffer (NucliSENS easyMag extraction buffer), mixed by gently pipetting up and down 20 times and incubated for 20 minutes at room temperature (21°C) before being stored at -80°C. Subsequently, 140 μL of this material was extracted into 60 μL elution buffer using a Qiagen viral RNA kit and stored at -70°C in 10 μL aliquots. For viral RNA quantification after extraction, a standard curve was generated from a 10-fold dilution series of the viral lysate product in a molecular grade deionized (DI) water and by using droplet digital PCR (ddPCR) method. For such purposes, the envelope protein (E) gene PCR was used in combination with the one-step reverse transcription (RT)-ddPCR kit for the probes (BioRad, USA) and the QX200 droplet digital PCR machine (BioRad, USA). After measurements, a viral RNA amount of 2.28×10^7 copies/μL was found for the extracted viral lysate product. The viral lysate was also used for whole genome sequencing, which revealed that the SARS-CoV-2 virus was of global lineage B.1 and UK lineage UK109. The extracted RNA solution was further diluted (1 : 10) in Takara Bio EASY dilution buffer for stabilization and to prepare stock solution (2.28×10^6 copies/μL) for the electrochemical measurements. The electrochemical impedance spectroscopy (EIS) measurements of SARS-CoV-2 sample using different single-valent and multi-valent probes were performed after diluting the stock solution in two different proportions; 1 : 2.5 and 1 : 4.8 to prepare two working concentrations of 9.09×10^5 copies/μL and 4.74×10^5 copies/μL, respectively using nuclease-free DI water and EIS buffer. The RNA dilution buffer (Takara Bio EASY dilution solution) and viral transport media (Remel MicroTest M4RT) were also diluted (1 : 2.5) individually with nuclease-free DI water and EIS buffer and used as negative controls during SARS-CoV-2 sample measurements. Additionally, we collected 10 clinical samples from COVID-19 patients and used same methodology (inactivation and viral RNA extraction) for direct analysis with our EIS biosensors. Prior to the EIS measurements, the samples were further diluted (1 : 2.5) with nuclease-free DI water and EIS measurement buffer.

Statistics. Statistical significance was determined using a 2-way ANOVA test, except in **Figure 4A** (COVID-19 patient sample analysis) in main article. An unpaired t test was used in **Figure 4A** to determine the statistical significance. The p value of 0.05 was set as threshold and the symbols ****, ***, **, * and ns represent $p \leq 0.0001$, $p \leq 0.001$, $p \leq 0.01$, $p \leq 0.05$ and $p > 0.05$, respectively as per the conventional practice.

Supplementary Results Section

Optimal Single-valent Probe Concentrations. To investigate the behavior of each single-valent probe individually and to determine the optimal condition for their individual performance, we varied the concentrations of the three single-valent probes (P-N1, P-RdRp1 and P-RdRp2) in the solution that was used for electrode functionalization. We observed an overall steady signal increase for P-N1 from 1.5 to 27 μM (**Figure S1A**), with a reproducible local minimum at 15 μM. For P-RdRp1 and P-RdRp2, the probes showed an almost constant, overall lower, signal intensity between 1.5 to 9 μM (**Figure S1B** and **S1C**).

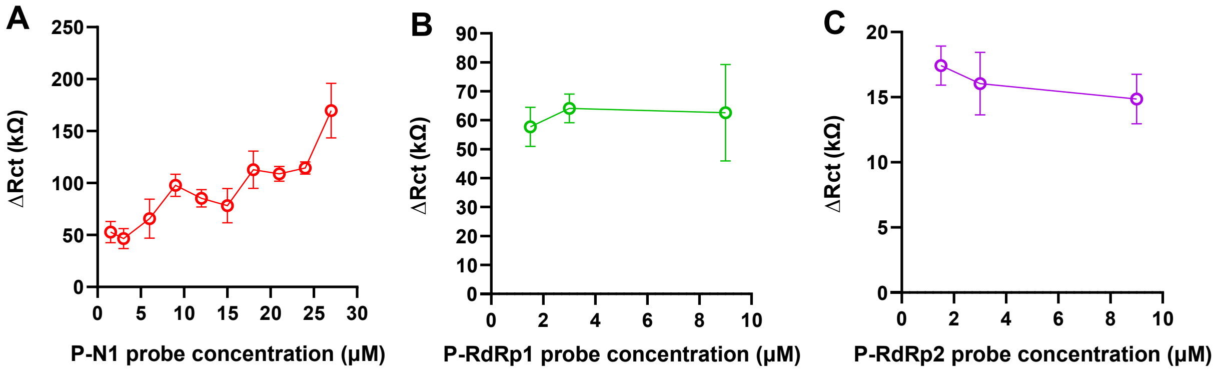

Figure S1. Effect of individual single-valent probe concentration variation: EIS signals (ΔRct) of electrodes functionalized with individual (A) P-N1 (1.5 to 27 μM), (B) P-RdRp1 (1.5 to 9 μM) and (C) P-RdRp2 (1.5 to 9 μM) probes, for different concentrations of probe in the solution used for electrode functionalization. All measurements were taken after 35 min hybridization with respective complementary DNA targets (50 nM). Data represent the mean ± SD; n ≥ 3.

Co-immobilization Causes Response Suppression. Next, we combined two or three of the single-valent probes together, with the aim of achieving multi-valent binding of the SARS-CoV-2 target. We first varied the probe concentration (1.5 to 9 μM for each probe) in equimolar mixtures of 2 or 3 probes, and tested them with the respective mixture of complementary DNA targets (50 nM each). We observed that the signals from the probe mixtures were almost independent of probe concentration (**Figure S2B** and **S2C**). In contrast to the expectation of an additive behavior, the signal obtained for the probe mixtures was suppressed compared to that obtained for the individual probes (**Figure S2A**). This effect was stronger for three probes than for dual combinations of the probes. The same pattern was observed for all three concentrations (1.5, 3 and 9 μM).

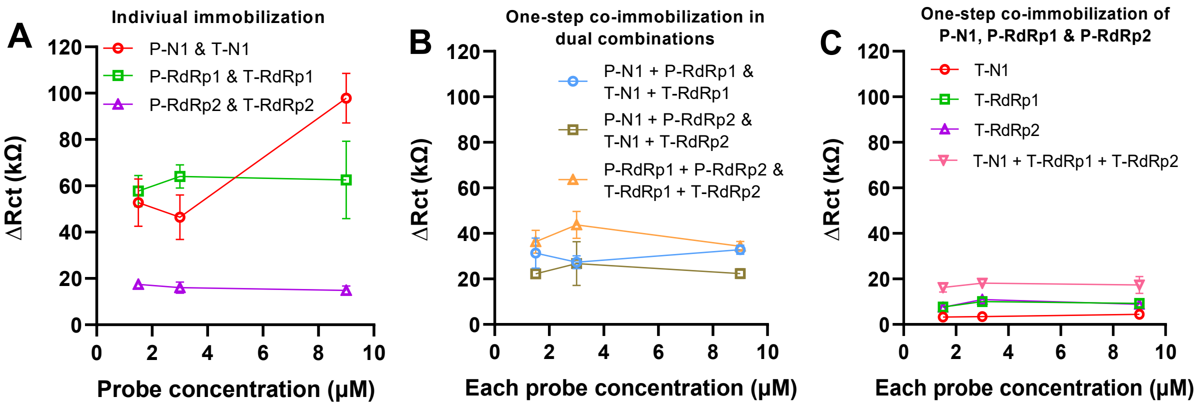

Figure S2. Effect of probe concentration, probe combination, and immobilization sequence on signal intensity: EIS signals (ΔRct) of electrodes functionalized with P-N1, P-RdRp1 and P-RdRp2 probes (1.5 to 9 μM concentration range, all probes in equimolar concentration), (A) immobilization of single probes, (B) one-step co-immobilization of two probes and (C) one-step co-immobilization of three probes. All measurements were taken after 35 min complementary DNA target (50 nM each) hybridization. When three probes were used together, individual target (50 nM) measurements were also taken. Data represent the mean ± SD; n = 4.

We considered that the response suppression could be occurring at late times, when the response kinetics starts to saturate. To investigate this, we also recorded the EIS response a shorter time (10 min) after hybridization with the DNA targets (**Figure S3A** and **S4B**). At this time, the EIS response has not yet started to saturate. However, we still observed significant suppression for the response of co-immobilized probes at the shorter (10 min) time (**Figure S3B** and **S4C**). To quantify the suppression, we defined a `suppression factor'. If there were no interaction between the probes, we would expect the response for co-immobilized

probes to equal the sum of the individually-immobilized responses for the same set of probes. We defined this sum as the `expected co-immobilized response', and defined the suppression factor as the ratio (expected co-immobilized response)/(measured co-immobilized response). A large value of the suppression factor implies strong suppression of the response due to probe-probe interactions, while a suppression factor value of 1 implies an absence of suppressive probe-probe interactions. For our datasets, the suppression factor varied among the different probe combinations, and was generally higher when 3 probes were co-immobilized compared to experiments where 2 probes were co-immobilized (**Figure S3B** and **S4C**).

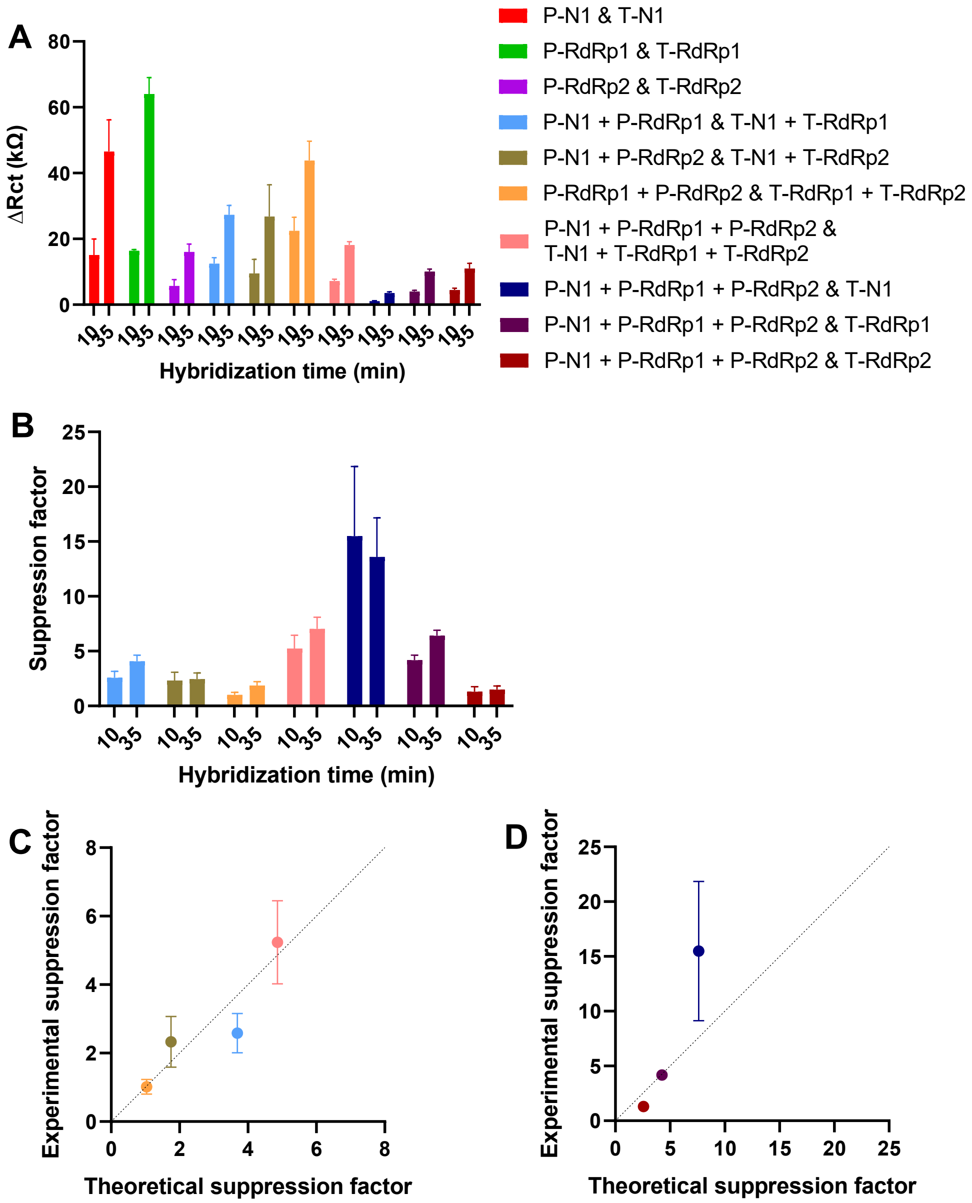

Figure S3. Influence of multiple single-valent probe combinations on sensor performance at room temperature (21°C): (A) EIS signal (ΔRct) comparisons for multiple probe combinations after 10 and 35 min hybridization at 3 μM concentrations of P-N1, P-RdRp1 and P-RdRp2 and 50 nM complementary target concentrations each, (B) suppression of signals for dual and triple probe combinations, (C) correlation between experimental suppression factor and theoretically predicted suppression factor using 10 min hybridization data for complementary targets and (D) same as C but for triple probes with single targets. Legend applies to all subfigures. Data represent the mean ± SD; n = 4.

Inter- and Intra-Probe Hybridization Can Explain Response Suppression. We hypothesized that the response suppression might be caused by inter- or intra-probe hybridization. To check this, we used NuPack to estimate the free energies of all possible inter- and intra-probe hybridization states (see **Supplementary Experimental Section**). Using these free energy values, we could calculate the theoretical equilibrium concentration of unhybridized probe monomers (i.e. those without internal secondary structure or hybridization to other probes), for a given mixture of probes (see **Supplementary Experimental Section**). Assuming that only unhybridized probe monomers are available to bind to the target and hence to contribute to the response, we could then predict theoretically the suppression factor for co-immobilized probes (see **Supplementary Experimental Section**). The theoretically-predicted suppression factors were in good agreement with our measured values, for the datasets measured 10 min after target hybridization (**Figure S3C** and **S4D**). The theoretical predictions did not agree well with our data measured after 35 min of target hybridization; this is not surprising since the theory does not account for the late-time response saturation.

Temperature Dependence of Single-valent Probes Binding to Size-matched DNA Targets. We anticipated that higher temperature measurements would reduce secondary structure formation and thus improve the hybridization signal in the combined probe experiments. Hence, we compared the EIS responses at room temperature (21°C) and 50°C for all probe combinations, at a specified probe concentration (3 μM). For both individual probes and probe combinations, we found a significant enhancement of the EIS signals at the higher temperature compared to room temperature, except for the P-RdRp2 probe immobilized individually (**Figure S4A**).

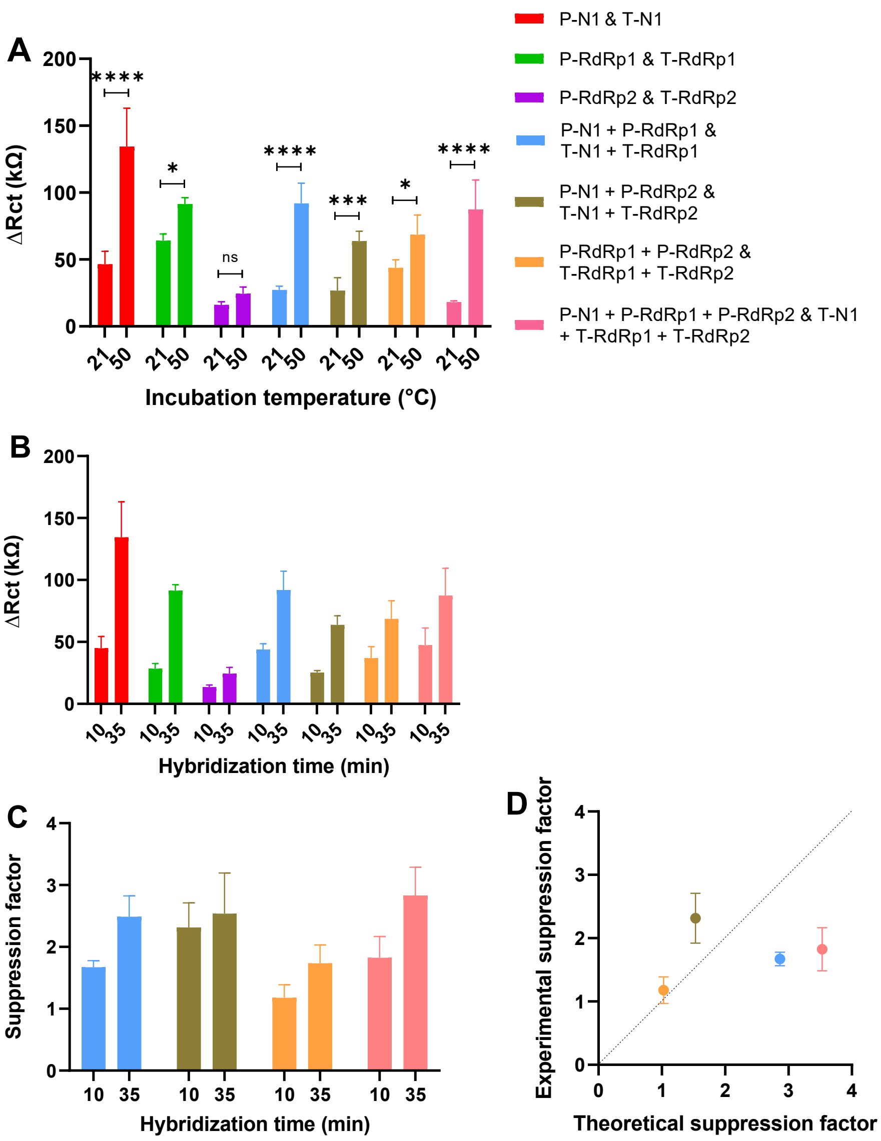

Figure S4. Effect of hybridization temperature on sensor performance: (A) EIS signals (ΔRct) of electrodes functionalized with P-N1, P-RdRp1 and P-RdRp2 probes (3 μM concentration each) with individual, dual and triple combinations at room temperature (21°C) or 50°C after 35 min complementary DNA target (50 nM each) hybridization, (B) EIS signal (ΔRct) comparisons for multiple probe combinations after 10 and 35 min hybridization, (C) suppression of signals for dual and triple probe combinations and (D) correlation between experimental suppression factor and theoretical suppression factor using 10 min hybridization data. Legend applies to all subfigures. Data represent the mean ± SD; n = 4.

Response suppression for co-immobilized probes was still present at the higher temperature (**Figure S4B** and **S4C**). Repeating our theoretical analysis at the higher temperature, we found less good agreement between theory and data than at room temperature (**Figure S4D**). This is expected since intra- and inter-probe hybridization is reduced with increasing temperature.

Based on these results, we concluded that the P-N1 + P-RdRp1 combination showed the most promising characteristics for multi-valent binding of the SARS-CoV-2 target. In particular, this probe combination showed strong responses to the DNA targets, as well as significant temperature enhancement (p < 0.0001). The P-N1 + P-RdRp1 + P-RdRp2 combination also produced similar results, but we suspected that this was

mainly due to the P-N1 and P-RdRp1, since we did not see a significant signal increase upon adding the P-RdRp2, compared to the P-N1 + P-RdRp1 combination.

Performance of Multi-valent Probes with Size-matched DNA Targets. We verified the performance of each multi-valent probe (P-MV1, P-MV2, and P-MV3) at the same probe concentration (6 µM) and with the respective complementary DNA targets (50 nM), using EIS measurements. With the exact size-matched DNA targets, all three multi-valent probes showed a significant response (**Figure S5**). The relative measured signal strength for the multi-valent probes was in the order; P-MV2 > P-MV1 > P-MV3.

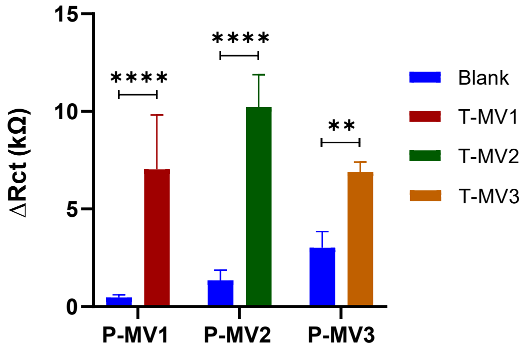

Figure S5. Multi-valent probe-to-probe comparison: EIS signals (ΔRct) of electrodes functionalized with 6 µM concentration of P-MV1, P-MV2 and P-MV3 after 35 min incubation with the respective complementary, size-matched DNA targets at 50 nM concentration. Data represent the mean ± SD; n ≥ 3.

Supplementary Table, Scheme and Figures

Table S1. Nomenclature and sequence of PNA probes and DNA targets used in present study

Sequence name	Type	Probe sequence (N-C)<br>Target sequence (5'-3')	Length (nt)	PNA N-end modification	PNA C-end modification	SARS-CoV-2 genome region (NC_045512)	SARS-CoV-2 genome position (bp)
P-N1	PNA	ATT TCG CTG ATT TTG GGG TC	20	Thiol-C11-AEEEA	None	Nucleocapsid protein (N) gene	28287 - 28306
P-RdRp1	PNA	CCG CCA CAC ATG ACC ATT TCA C	22	Thiol-C11-AEEEA	None	RdRp (ORF1b) gene	15431 - 15452
P-RdRp2	PNA	CAA ATG TTA AAA ACA CTA TTA GCA TA	26	Thiol-C11-AEEEA	None	RdRp (ORF1b) gene	15505 - 15530
P-RdRp3	PNA	GCA TCT CCT GAT GAG GTT CCA CCT G	25	Thiol-C11-AEEEA	None	RdRp (ORF1b) gene	15470 - 15494
P-MV1	PNA	TCG CGG GG	8	Thiol-C11-AEEEA	None		
P-MV2	PNA	CCT GAG GGA G	10	Thiol-C11-AEEEA	None		
P-MV3	PNA	GTA GCT CTT CGG T	13	Thiol-C11-AEEEA	None		
P-miR122	PNA	CAA ACA CCA TTG TCA CAC TCC A	22	Thiol-C11-AEEEA	None		
T-N1	DNA	GAC CCC AAA ATC AGC GAA AT	20				
T-RdRp1	DNA	GTG AAA TGG TCA TGT GTG GCG G	22				
T-RdRp2	DNA	TAT GCT AAT AGT GTT TTT AAC ATT TG	26				
T-RdRp3	DNA	CAG GTG GAA CCT CAT CAG GAG ATG C	25				
T-MV1	DNA	CCC CGC GA	8				
T-MV2	DNA	CTC CCT CAG G	10				
T-MV3	DNA	ACC GAA GAG CTA C	13				
MV3 RNA	RNA	ACC GAA GAG CUA C	13				
SARS-CoV-2	RNA		29903				

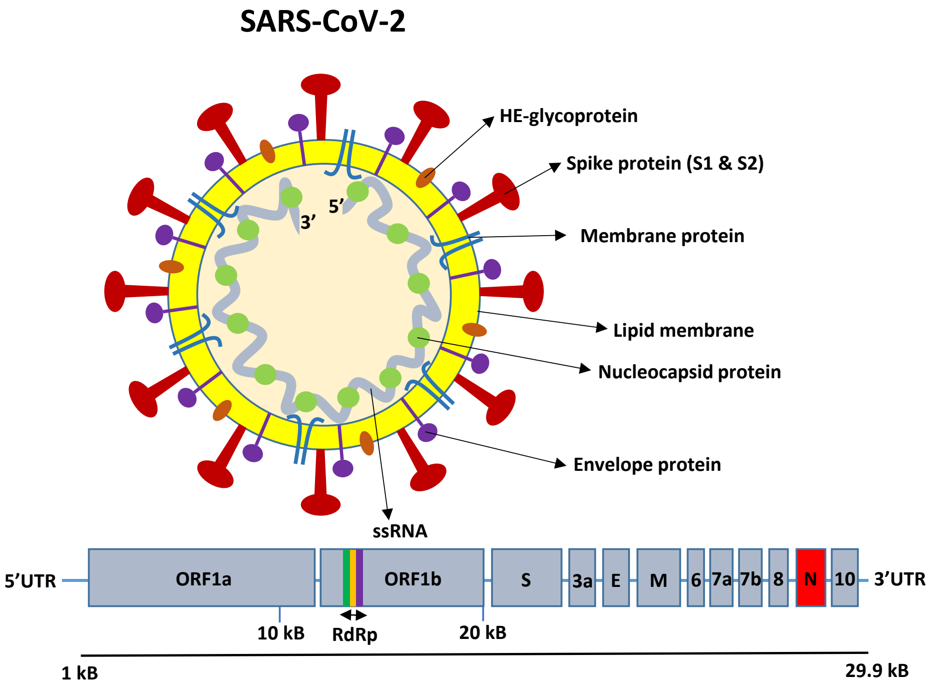

Scheme S1. SARS-CoV-2 structure and genome organization indicating structural features and target regions. RNA-dependent RNA polymerase (RdRp) and nucleocapsid protein (N) gene regions were used for designing single-valent probes.

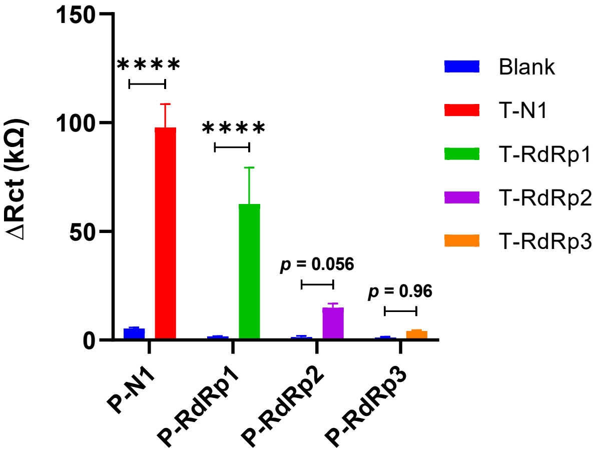

Figure S6. Single-valent probe-to-probe comparison: EIS signals (ΔRct) of electrodes functionalized with 9 µM of P-N1, P-RdRp1, P-RdRp2 and P-RdRp3 probes after 35 min incubation with 50 nM complementary DNA targets. Data represent the mean ± SD; n = 4.

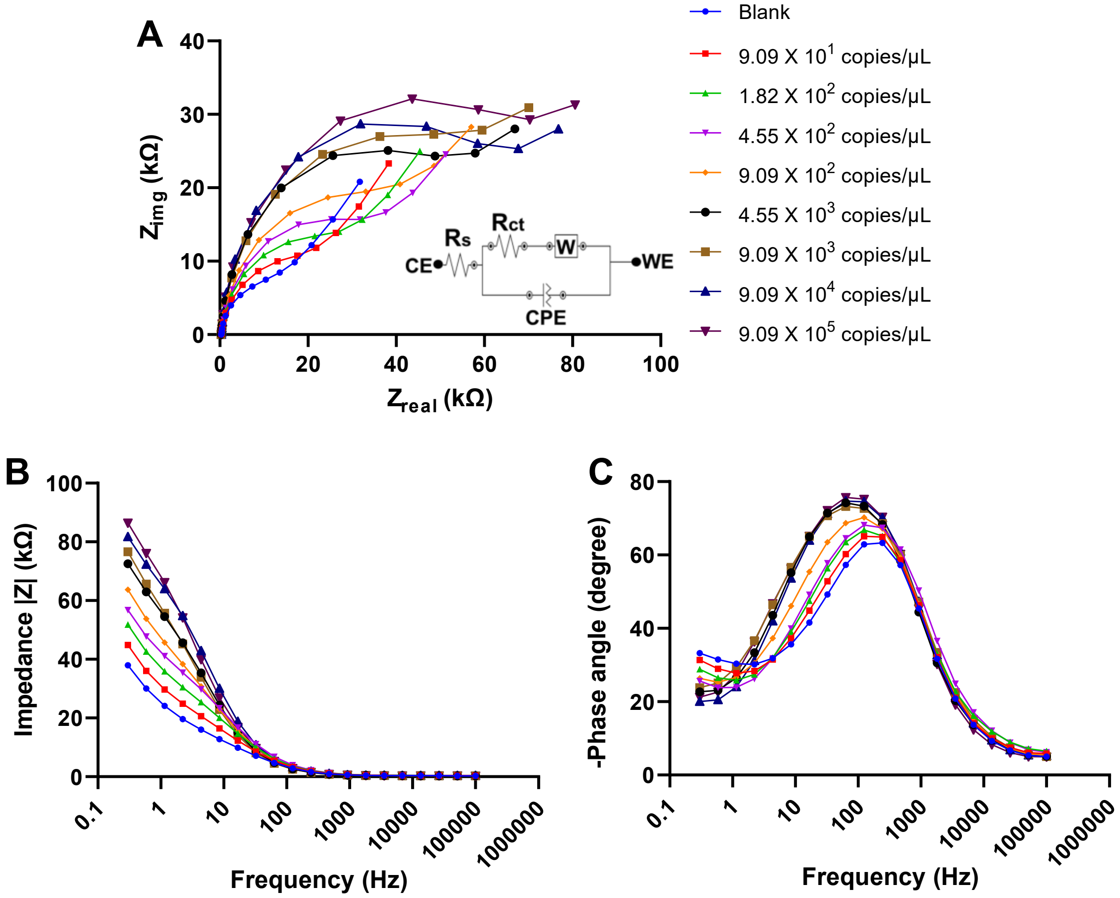

Figure S7. EIS signals of electrodes functionalized with the combination of P-N1 and P-RdRp1 (3 µM each) after 35 min incubation at 50°C with buffer control (blank) and SARS-CoV-2 RNA target (9.09×10^1 - 9.09×10^5 copies/µL); (A) Nyquist plots with the equivalent Randles' circuit. CE, WE, Rs, Rct, W and CPE represent working electrode, counter electrode, solution resistance, charge transfer resistance, Warburg element and constant phase element, respectively, (B) Bode plots showing magnitude of impedance as a function of frequency and (C) Bode plots showing phase angle shift as a function of frequency. Legend applies to all subfigures.

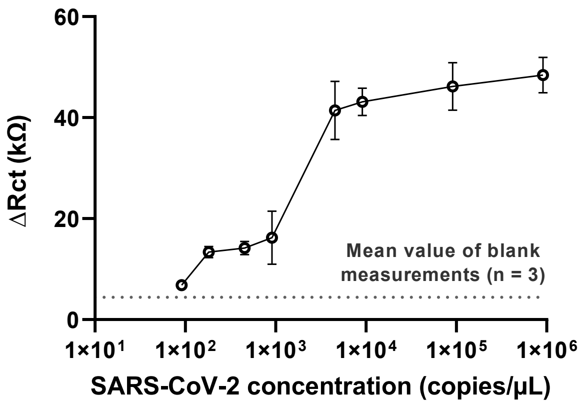

Figure S8. Dose dependent direct detection of SARS-CoV-2 RNA: EIS signals (ΔRct) of electrodes functionalized with the combination of P-N1 and P-RdRp1 (3 µM each) after 35 min incubation at 50°C with SARS-CoV-2 RNA target (9.09×10^1 - 9.09×10^5 copies/µL). The dotted line represents blank measurements (mean value 4.45 kΩ and SD ± 1.52 kΩ). Data represent the mean ± SD; n = 3.

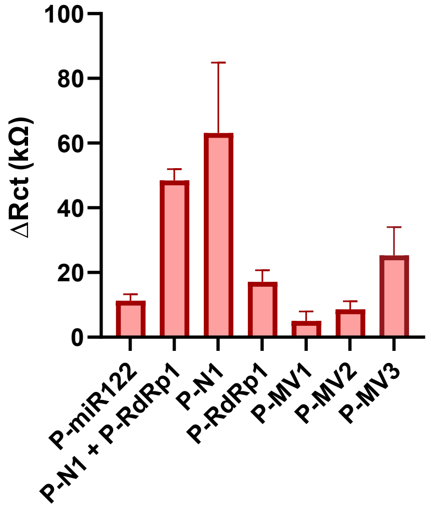

Figure S9. Comparison of specific probe or probe combination with a negative control probe for SARS-CoV-2 RNA detection: EIS signals (ΔRct) of electrodes functionalized with specific probes P-N1 (3 μM), P-RdRp1 (3 μM), combination of P-N1 and P-RdRp1 (3 μM each), P-MV1 (6 μM), P-MV2 (6 μM) and P-MV3 (6 μM) or negative control probe P-miR122 (6 μM) after 35 min incubation at 50°C with SARS-CoV-2 RNA sample (9.09×10^5 copies/μL). Data represent the mean ± SD; n = 3.

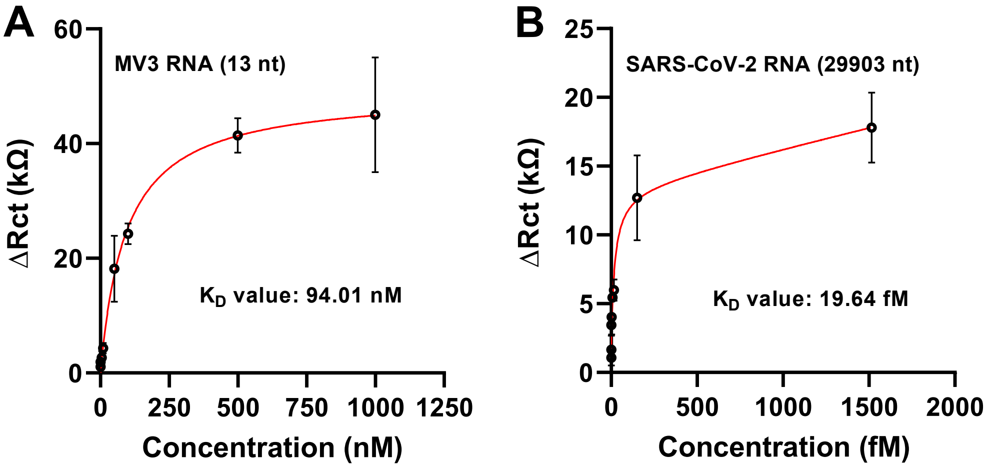

Figure S10. Dose dependent target detection for analyzing multi-valency of P-MV3 probe: EIS signals (ΔRct) of electrodes functionalized with P-MV3 (6 μM) after 35 min incubation at room temperature (21°C) with (A) size-matched RNA oligo (13 nt) (1 – 1000 nM), and (B) full-length SARS-CoV-2 RNA target (29,903 nt) (0.76 fM – 1.52 pM). All data represent the mean ± SD; n = 3. Nonlinear curve fittings have been done using the model $Y = Bmax*X/(K_D + X)$, where Bmax is the maximum specific binding (kΩ) and K_D is the equilibrium dissociation constant (nM in panel A or fM in panel B).

References

1. Zadeh, J. N.; Steenberg, C. D.; Bois, J. S.; Wolfe, B. R.; Pierce, M. B.; Khan, A. R.; Dirks, R. M.; Pierce, N. A., NUPACK: Analysis and design of nucleic acid systems. *J. Comput. Chem.* **2011,** *32* (1), 170-173.
2. Curk, T.; Brackley, C. A.; Farrell, J. D.; Xing, Z.; Joshi, D.; Direito, S.; Bren, U.; Angioletti-Uberti, S.; Dobnikar, J.; Eiser, E.; Frenkel, D.; Allen, R. J., Computational design of probes to detect bacterial genomes by multivalent binding. *Proc. Natl. Acad. Sci. U.S.A.* **2020,** *117* (16), 8719-8726.
3. Egholm, M.; Buchardt, O.; Christensen, L.; Behrens, C.; Freier, S. M.; Driver, D. A.; Berg, R. H.; Kim, S. K.; Norden, B.; Nielsen, P. E., PNA hybridizes to complementary oligonucleotides obeying the Watson–Crick hydrogen-bonding rules. *Nature* **1993,** *365* (6446), 566-568.
